# The Impact of Convertase Subtilisin/Kexin Type 9 Monoclonal Antibodies with and without Apheresis on Platelet Aggregation in Familial Hypercholesterolemia

**DOI:** 10.1007/s10557-023-07455-y

**Published:** 2023-05-02

**Authors:** Lukáš Konečný, Marcel Hrubša, Jana Karlíčková, Alejandro Carazo, Lenka Javorská, Kateřina Matoušová, Lenka Kujovská Krčmová, Vladimír Blaha, Milan Bláha, Přemysl Mladěnka

**Affiliations:** 1 The Department of Pharmacology and Toxicology, Faculty of Pharmacy in Hradec Králové, Charles University, 50005 Hradec Králové, Czechia; 2 The Department of Pharmacognosy and Pharmaceutical Botany, Faculty of Pharmacy in Hradec Králové, Charles University, 50005 Hradec Králové, Czechia; 3 The Department of Clinical Biochemistry and Diagnostics, University Hospital Hradec Králové, 50005 Hradec Králové, Czechia; 4 The Department of Analytical Chemistry, Faculty of Pharmacy in Hradec Králové, Charles University, 50005 Hradec Králové, Czechia; 5 The 3rd Department of Internal Medicine-Metabolic Care and Gerontology, University Hospital and Faculty of Medicine in Hradec Králové, Charles University, 50005 Hradec Králové, Czechia

**Keywords:** Dyslipidemia, Antiplatelet, Acetylsalicylic acid, ADP receptor, Ticagrelor, Vorapaxar

## Abstract

**Background and aims:**

It is well known that elevated cholesterol is associated with enhanced platelet aggregation and patients suffering from familial hypercholesterolemia (FH) have a high risk of thrombotic cardiovascular events. Although decreasing cholesterol level is associated with attenuation of platelet hyperactivity, there are currently no data on the effect of convertase subtilisin/kexin type 9 monoclonal antibodies (PCSK9ab) on platelet reactivity in FH. The aim of the study was to analyse the impact of different therapies including PCSK9ab on platelet aggregation in FH.

**Methods:**

This study enrolled all 15 patients treated in the University Hospital Hradec Králové for FH. PCSK9ab have been administered in 12 of 15 patients while 8 patients were also undergoing lipid apheresis. Blood samples from all patients including pre- and post-apheresis period were tested for platelet aggregation triggered by 7 inducers, and the effect of 3 clinically used drugs (acetylsalicylic acid, ticagrelor and vorapaxar) was compared as well.

**Results:**

Although apheresis decreased the reactivity of platelets in general, platelet responses were not different between non-apheresis patients treated with PCSK9ab and apheresis patients (post-apheresis values) with the exception of ristocetin. However, when compared to age-matched healthy population, FH patients had significantly lower platelet aggregation responses to 4 out of 7 used inducers and higher profit from 2 out of 3 used antiplatelet drugs even after exclusion of FH patients regularly receiving conventional antiplatelet treatment.

**Conclusion:**

This study showed for the first time the suitability of PCSK9ab treatment for reduction of platelet reactivity in FH patients.

**Supplementary Information:**

The online version contains supplementary material available at 10.1007/s10557-023-07455-y.

## Introduction

There is no doubt that enhanced low-density lipoprotein cholesterol (LDL-C) level is one of the main risk factors for atherosclerosis leading to coronary artery disease. Indeed, patients suffering from familial hypercholesterolemia (FH) die from cardiovascular disease prematurely. FH is an inherited disease causing elevated levels of LDL-C. A causal relationship between elevated LDL-C and hyperaggregability is known as well and it can be assumed that increased platelet function depends on the higher amount of circulating lipoproteins with atherogenic apolipoprotein B 100 (ApoB100) on their surface [1]. FH is mainly caused by one of four basic mutations. A mutation in the gene for the LDL receptor (LDL-R) is detected most often (90% of cases). This mutation prevents LDL-C from binding to their receptor because there are few or no LDL-R. Other common mutations include ApoB100, proprotein convertase subtilisin/kexin type 9 (PCSK9) or LDL-R adaptor protein (LDL-RAP1). ApoB100 is needed for interaction of LDL-C with the LDL receptor. PCSK9ab is responsible for LDL-R degradation and its overexpression is hence associated with more extensive degradation of LDL-R [2]. LDL-RAP1 collaborates with LDL-R and enables endocytosis of LDL-C after binding to that receptor and hence mutation of the LDL-RAP1 gene (autosomal recessive disease) leads to FH. Regardless, in all cases, LDL-C levels are markedly elevated in comparison with healthy population [3–5].

Current treatment possibilities are based on these pathophysiological issues. Statins are used as in other hypercholesterolemic patients, but their impact on the level of LDL-C is low in this case. For a long time, repeated lipid apheresis was the only reasonable choice for decreasing LDL-C levels in FH. Antisense nucleotide mipomersen against mRNA for ApoB100, approved in 2013, was one of the earliest targeted drugs for FH but its effect is burdened by potential hepatotoxicity. Finally, the development of PCSK9 monoclonal antibodies (PCSK9ab) brought a marked improvement in FH treatment. Most recently, a small interfering RNA against PCSK9 inclisiran was registered by both FDA and EMA [6]. As the drugs against PCSK9ab are relatively new, there is very limited information on their impact on platelet aggregation in contrast to many publications reporting increased platelet aggregation in FH and the positive effect of LDL-apheresis [7–9]. The last options for lipid-lowering therapy are (1) lomitapide as adjuvant therapy, for those patients who have not achieved LDL-C goals with apheresis and any other lipid modifying agent combination [10], or (2) the newest drug evinacumab approved in 2021 [11]. They might be less suitable due to possible occurrence of specific side effects, which encompass liver toxicity in the former and a decrease in HDL cholesterol in the latter case [12, 13]. The effect of inclisiran, lomitapide and evinacumab on LDL-C in homozygous FH is comparable and achieves a 50% reduction [10, 14].

Platelet aggregation is physiologically an essential part of haemostasis which is strictly regulated by a number of biological factors. It is known that pathological conditions such as atherosclerosis or inflammation lead to hyperactive platelets which are linked to an elevated risk of cardiovascular disease [15]. High levels of oxidized LDL-C trigger platelet aggregation after binding to their specific receptors on thrombocyte surface, mainly scavenger receptor CD36. This cascade is associated with activation of cytosolic phospholipase A_2_ which releases arachidonic acid (AA). AA is then enzymatically transformed to thromboxane A_2_ by cyclooxygenase 1 and thromboxane synthase. The process is associated with enhanced cytosolic Ca^2+^ content and inhibition of nitric oxide release. Nitric oxide has important antiplatelet effects and hence it is not surprising that platelet aggregation is enhanced in FH [1, 7, 15–18].

In this study, we aimed (1) to examine the effect of treatment by monoclonal antibodies against PCSK9 on different aggregatory cascades using 7 platelet aggregation triggers as well as on the antiplatelet potency of three clinically used drugs in FH patients, and (2) to compare platelet responses to these triggers and antiplatelet drugs between FH patients and age-matched generally healthy controls.

## Materials and Methods

### Donors

**Fig. 1:**
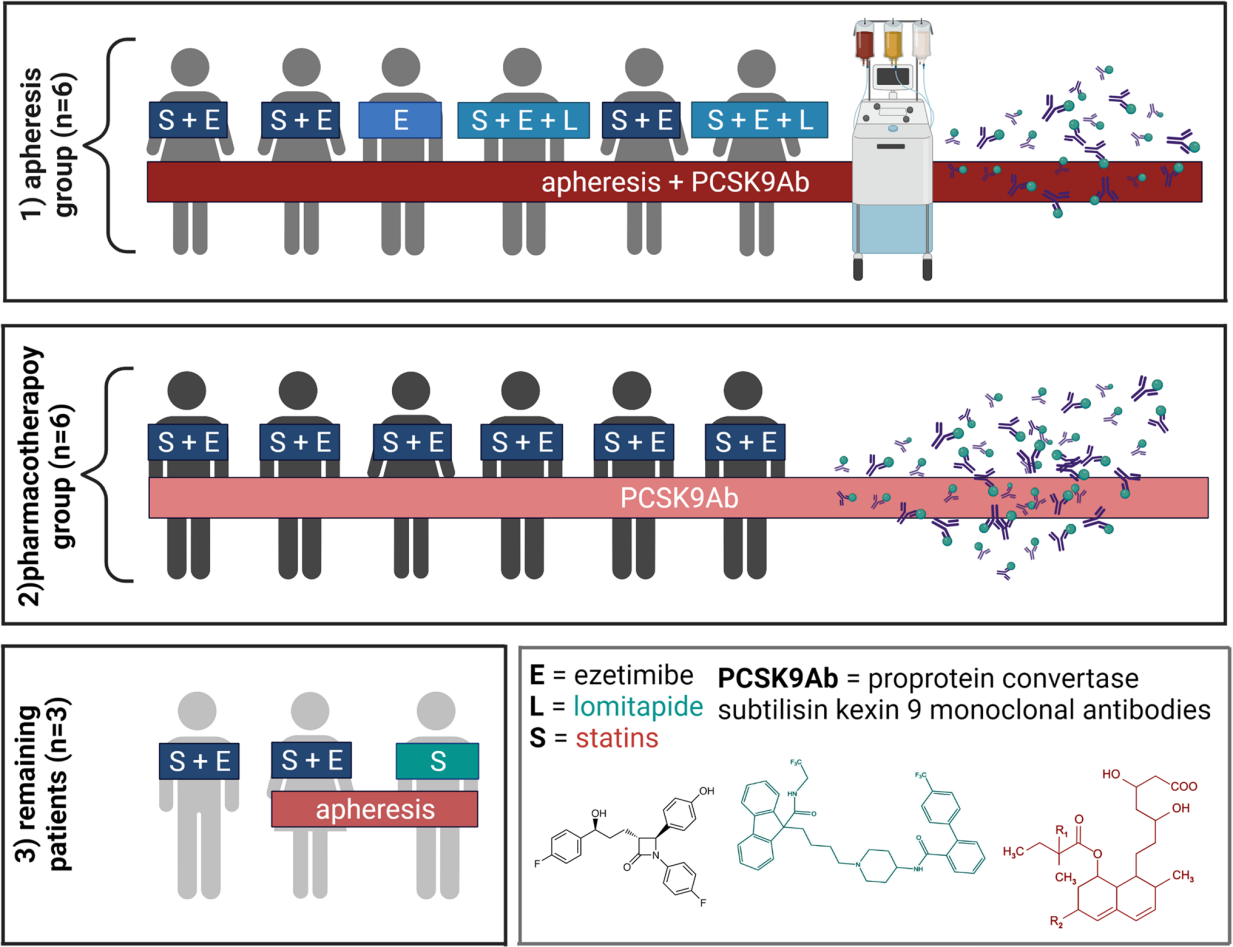
Included familial hypercholesterolemia (FH) patients, their treatment and subgroups. For reason of comparison between FH treatment modalities, patients were divided into 2 subgroups — (1) apheresis group (*n* = 6) and (2) pharmacotherapy group (*n* = 6). The remaining 3 FH patients were used solely together with other 12 patients from the above-mentioned two subgroups for comparison between FH and age-matched generally healthy controls

Blood was collected from 15 patients suffering from familial hypercholesterolemia, 4 homozygous patients (HoFH) and 11 heterozygous patients (HeFH) in the University Hospital Hradec Králové. These 15 patients represent all FH patients of this hospital which treats FH patients coming from all parts of Czechia. The novel treatment with PCSK9ab was used in 12 of 15 patients. Moreover, 8 patients were also undergoing lipid apheresis. In the apheresis group, 6 patients were also treated with PCSK9ab while the remaining two with other hypolipidemic drugs following the current guidelines. In the non-apheresis group (pharmacotherapy group), 6 out of 7 patients were treated with PCSK9ab. For the purposes of this study, the effect of apheresis was compared in all patients treated with this modality. The effect of PCSK9ab was compared between patients treated with and without apheresis. Moreover, the data on all FH patients were compared to an age-matched controls which were selected from our previous study [19]. Fig. 1 summarizes all included FH patients and their hypolipidemic treatment. Their detailed characteristics are further specified in the Supplementary information Tables S1–S6. All donors signed an informed consent in line with the approval of the ethics committee of the University Hospital in Hradec Králové (No. 202007 S01P from June 18th, 2020). All experiments conformed to the latest Declaration of Helsinki.

### Blood Collection

Drugs known to affect platelet aggregation (e.g., non-steroidal anti-inflammatory drugs) were not allowed 24 h prior to blood collection. Also, alcohol was not allowed as it has an effect on platelet aggregation. Blood samples were collected by venepuncture into plastic disposable syringes containing either heparin sodium (17 IU/mL, for platelet aggregation experiments) or a clotting accelerator (for making biochemical assessment of basic metabolic parameters in serum). The collections were performed in the morning at 8–9 a.m. and the donors also brought morning urine samples. In the case of post-apheresis samples, the blood was collected after the procedure (approximately 4 h after the pre-apheresis samples).

### Lipid Apheresis

As already mentioned, apheresis was performed in 8 FH patients. The aim of this method is the extracorporeal elimination of blood cholesterol using special adsorption columns or filters. The procedures were performed from the peripheral vein in the elbow pit or in the forearm. Plasma separation was performed using a Cobe-Spectra or Optia continuous centrifugal separator (Terumo, Likewood, CO, USA). Washed plasma with erythrocytes was returned to another peripheral vein. The process resulted in purification of 100–150% of the volume of circulating plasma. A mixture of heparin and citrate dextrose solution A (Baxter, Munich, Germany) were used as anticoagulant. Three different apheresis principles were used in these patients. In the case of 3 patients, immunoadsorption Lipopak adsorber column (Pocard, Moscow, Russia) employing sheep antibodies against apolipoprotein B 100 was used. In the other 2 patients, Lipocollect column (Medicollect, Ullrichstein, Germany) was used. In these 5 patients, the flow of plasma was controled by Adasorp (Ullrichstein, Germany). In the remaining 3 FH patients Evaflux 4A filter (Kawasumi, Tokyo, Japan) was used since they also had hyperfibrinogenemia. This method called rheohaemapheresis was used according to Borberg et al. with our own modifications [20, 21]. The flow through the filter was controlled using the CF100 automatic machine (Infomed, Geneva, Switzerland).

### Chemicals

Dimethyl sulfoxide (DMSO); acetylsalicylic acid (ASA); ticagrelor; ristomycin monosulfate (ristocetin); platelet activating factor-16 (PAF) and 9,11-dideoxy-11α,9α-epoxymethanoprostaglandin F_2α_ (U-46619) were purchased from Sigma (St. Louis, MO, USA). Thrombin receptor agonist peptide-6 (TRAP), adenosine-5-diphosphate (ADP) and AA were purchased from Roche (Basel, Switzerland). Vorapaxar was purchased from Selleck Chemicals GmbH (Planegg, Germany). Collagen was purchased from Diagnostica, a.s. (Prague, Czechia) and 0.9% sodium chloride (saline) from B. Braun (Melsungen, Germany).

### Assessment of Platelet Aggregation

**Fig. 2:**
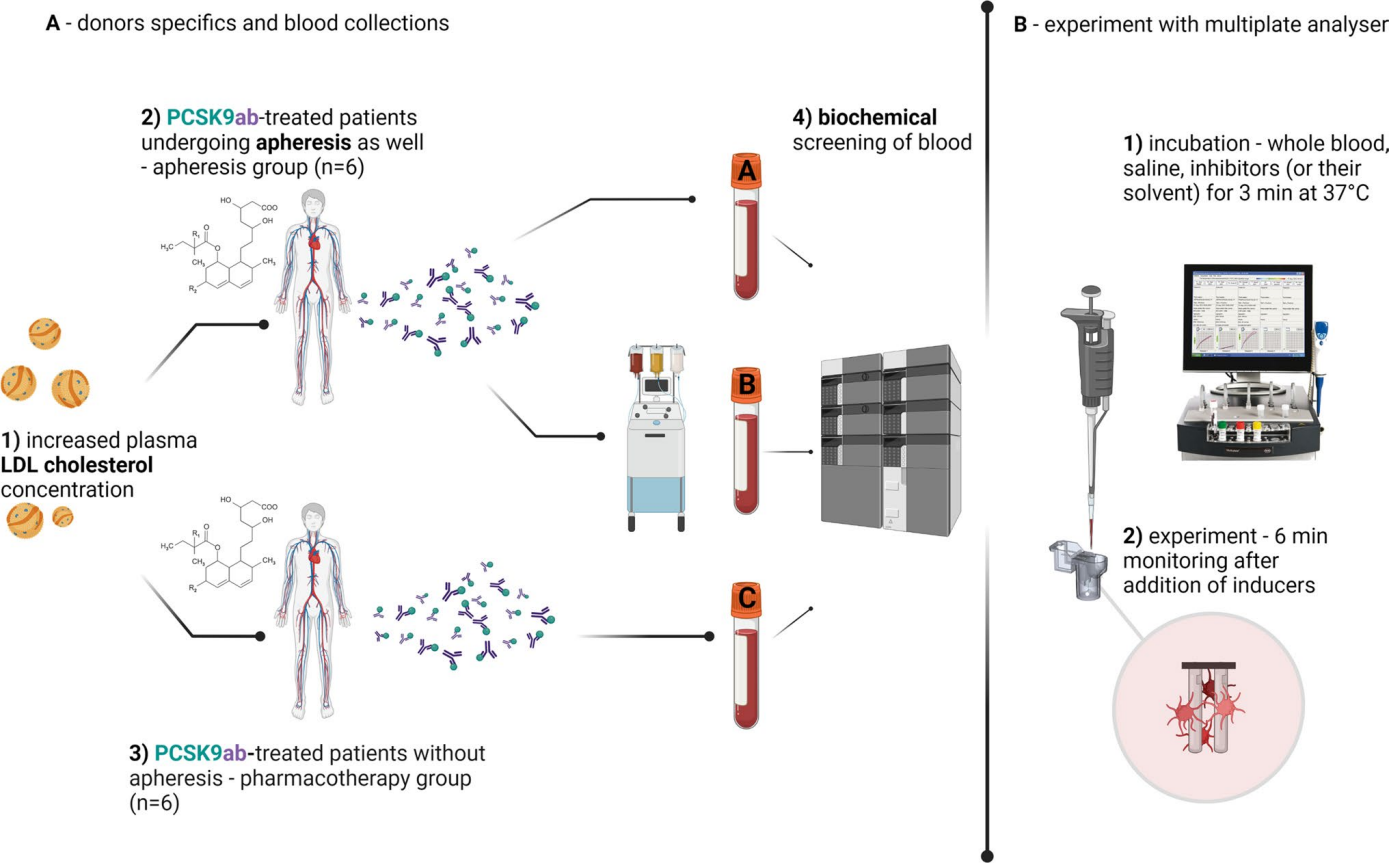
Graphical scheme of the performed experiment. The first part (A) depicts both treatment types and blood collection: **1** — patients suffering from familial hypercholesterolemia (FH); **2** — the first group of patients treated with PCSK9ab and undergoing apheresis as well (apheresis group, *n* = 6), the samples were measured before apheresis — tube A and after apheresis — tube B; **3** — the second group of patients treated with PCSK9ab (pharmacotherapy group, *n* = 6) — tube **C**; **4** — all samples (including the rest of FH patients, total *n* = 15) were tested for biochemical parameters (glucose, serum lipids and creatinine). The second part (B) depicts experiment with platelet aggregation analyser: **1** — experiment was initiated precisely 30 min after the blood draw, whole blood with preheated saline was incubated with tested antagonist/inhibitor (ASA, vorapaxar, ticagrelor) or their solvent DMSO; **2** — 6 min monitoring after addition of inducers of platelet aggregation (collagen, ristocetin, TRAP, ADP, AA, PAF, U-46619). The administered hypolipidemic drugs are summarized in the Fig. 1 and Supplementary information (Supplementary information Table S4)

The graphical scheme of the procedure, including aggregation experiments, is shown in Fig. 2. Briefly, 300 µL of whole blood was diluted with the same volume of preheated 0.9% saline solution and incubated with 5 µL of the tested inhibitor/antagonist (ASA, ticagrelor, vorapaxar) or the used solvent DMSO as blank at a final concentration of 0.8% for 3 min at 37 °C. Platelet aggregation was then induced with one of the following aggregating agents: collagen, AA, PAF, U-46619, ristocetin, ADP or TRAP at desired concentration(s) and monitored for 6 min. The final concentration(s) of the following inducers and inhibitors are given in the supplementary information (Supplementary information Table S7). The aggregation response was quantified using the AUC (area under the curve).

### Measurement of Biochemical Parameters

#### Glucose, Lipids and Creatinine

Glucose, total cholesterol (TC), HDL cholesterol (HDL-C), LDL-C and triglycerides (TG) in serum were measured using commercial enzymatic kits by Cobas 8000 system (Roche, Basel, Switzerland). Creatinine was determined in both serum and urine. Analysis was carried out using a Prominence LC 20 HPLC set with an SPD-M20A Shimadzu (Shimadzu, Kyoto, Japan) diode array detector. As the stationary phase, two monolithic columns RP-18e (4.6 mm × 50 mm, 3.0 mm × 100 mm) were connected in combination with a 15 mM phosphate buffer as the mobile phase. Creatinine was detected at 235 nm using diode array detection [22].

### Statistical Analysis

GraphPad Prism 9.3.1. (GraphPad Software, San Diego, CA, USA) was used for all data analysis. Data were firstly analysed using the Shapiro–Wilk test for confirming or rejecting the Gaussian distribution. Based on this, differences were analysed by the unpaired Student *t*-test or Mann–Whitney test (controls vs. FH patients) or ANOVA (different FH modalities) followed by paired or unpaired Student *t*-test or the Mann–Whitney or Wilcoxon matched-pairs signed-rank tests, respectively. Differences in biological parameters and donor characteristics were analysed by the Chi-square test or the unpaired Student *t*-test. Correlations were performed by the Pearson’s correlation test and if significant, linear regression was also performed.

## Results

### Effect of LDL-Apheresis in PCSK9ab-Treated Patients

Blood of all FH patients was tested for platelet responses to seven inductors of aggregation with various mechanisms of action together with three clinically used antiplatelet drugs (ASA, ticagrelor, vorapaxar).

In the first step, platelet aggregation in all 12 PCSK9ab-treated FH patients was analysed. Precisely half of these patients were also undergoing apheresis, while the second half was treated solely pharmacologically. In patients treated with apheresis, both pre- and post-apheresis aggregation data were obtained. As expected, apheresis significantly decreased TC, LDL-C, HLD-C and non-HDL cholesterol (non HDL-C) as well as TG. There were no differences in any mentioned lipidic parameters between solely pharmacologically treated patients and pre-apheresis values in the apheresis group. However, LDL-apheresis significantly reduced TC, LDL-C and non HDL-C as well as TG when compared to the solely pharmacologically treated patients (Supplementary information Figure S1).

**Fig. 3:**
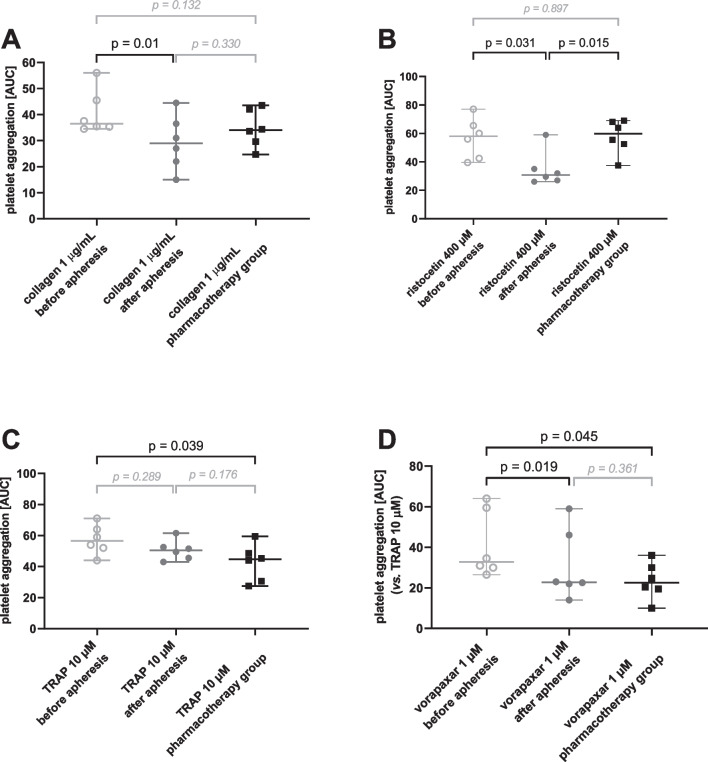
Comparison of various inducers of platelet aggregation with or without antagonist in all PCSK9ab-treated patients with or without lipid apheresis. **A** Area under the curve (AUC) of aggregation induced by collagen at a final concentration of 1 µg/ml in PSCK9ab-treated patients, with apheresis (before and after) and without apheresis. **B** AUC of aggregation induced by ristocetin at a final concentration of 400 µM in PSCK9ab-treated patients, with apheresis (before and after) and without apheresis**. C** AUC of aggregation induced by TRAP at a final concentration of 10 µM in PSCK9ab-treated patients, with apheresis (before and after) and without apheresis.** D** AUC of aggregation induced by TRAP after 1 µM vorapaxar pre-treatment in PSCK9ab-treated patients, with apheresis (before and after) and without apheresis. *P*-values were calculated by a paired test (pre- and post-apheresis) or an unpaired test (apheresis samples *vs.* pharmacotherapy group). Results are shown as median with 95% confidence interval

In general, LDL-apheresis reduced the reactivity of platelets to the inducers and improved the effect of antiplatelet drugs. A significant drop in platelet aggregation was observed for collagen and ristocetin (Figs. 3A, 3B) while insignificant tendencies were observed for the other inducers (AA, ADP, PAF, U-46619 and TRAP, Supplementary information Figure S2 and Fig. 3C). Similarly, the effect of vorapaxar on TRAP-triggered aggregation was significantly improved after apheresis (Fig. 3D). There was little difference between pre-apheresis values in the apheresis group and the pharmacotherapy group with one exception; TRAP induced lower platelet aggregation in the pharmacotherapy group when compared to pre-apheresis data. However, apheresis attenuated the TRAP response to values comparable to the pharmacotherapy group (Fig. 3C). There were no differences between pre, post-apheresis values and the pharmacotherapy group in the antiplatelet effects of ASA and ticagrelor.

### Comparison Between FH-Patients and Age-Matched Healthy Controls

**Fig. 4:**
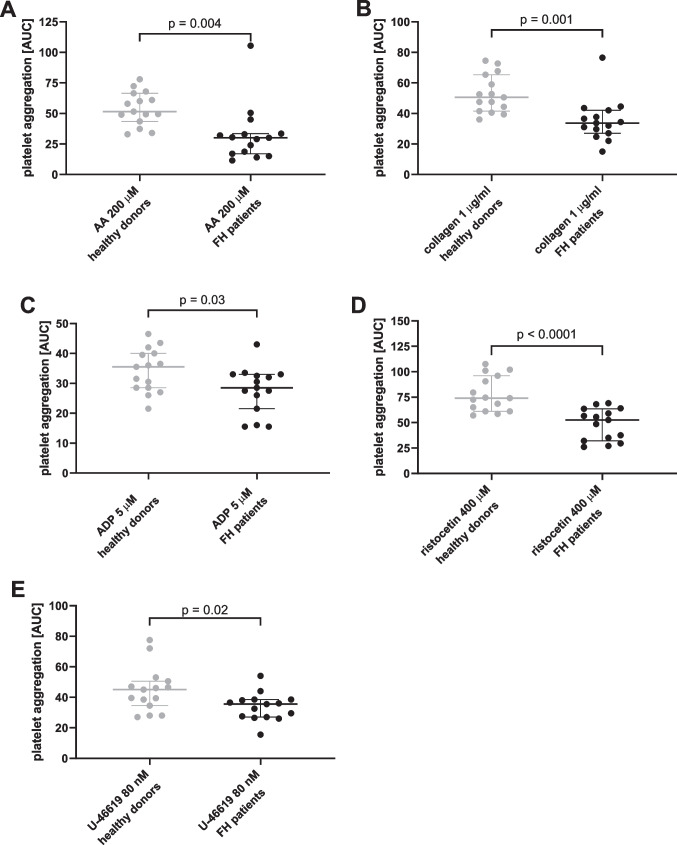
Comparison of various inducers of platelet aggregation in a group of generally healthy donors and all included familial hypercholesterolemic patients. **A** Area under the curve (AUC) of aggregation induced by AA at a final concentration of 200 μM.** B** AUC of aggregation induced by collagen at a final concentration of 1 µg/ml. **C** AUC of aggregation induced by ADP at a final concentration of 5 µM.** D** AUC of aggregation induced by ristocetin at a final concentration of 400 µM. **E** AUC of aggregation induced by U-46619 at a final concentration of 80 nM. *P*-values were calculated by an unpaired test. Results are shown as median with 95% confidence interval

**Fig. 5:**
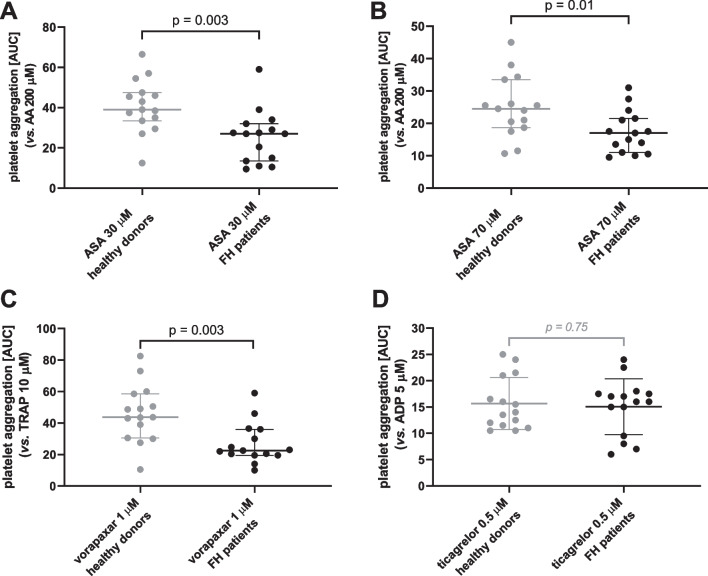
Effect of antiplatelet drugs on platelet aggregation in group of generally healthy donors and all included familial hypercholesterolemia patients. **A** AUC of aggregation induced by AA in blood pre-treated with 30 µM ASA.** B** AUC of aggregation induced by AA in blood pre-treated with 70 µM ASA. **C** AUC of aggregation induced by TRAP in blood pre-treated with 1 µM of vorapaxar. **D** AUC of aggregation induced by ADP in blood pre-treated with 0.5 µM of ticagrelor. *P*-values were calculated by an unpaired *t*-test. Results are shown as median with 95% confidence interval

The effect of hypolipidemic treatments (all 15 FH patients) was compared to an age-matched healthy control group. Healthy controls aggregated more strongly in response to all 7 used platelet aggregation inducers; 5 of them reached significant differences (Fig. 4). Moreover, inhibitory effect of ASA and vorapaxar was stronger in FH patients, while the effect of ticagrelor remained unchanged (Fig. 5). As 7 patients from the FH treated group were regularly administered with antiplatelet drugs (Supplementary information Table S2) and such treatment could not be discontinued since it was indicated for secondary prevention of cardiovascular events, it was necessary to perform additional analysis in order to prevent possible bias on the outcomes. After removal of these 7 patients, sensitivity of the analysis was lower, but the controls still reacted more strongly to AA, collagen, ristocetin and TRAP. There was also a tendency in the case of U-46619 (*p* = 0.053, Supplementary information Table S8). Two of these seven patients treated with antiplatelet drugs were, however, treated with enteric-coated ASA, which is currently not considered to be associated with biologically relevant antiplatelet effect [23–25]. For this reason, these 2 patients were re-included in the analysis and the results encompassing 10 patients were principally similar to those observed with all 15 FH patients (Figs. 4–5, Supplementary information Table S8). The only exception was that the difference in ADP-response did not reach a significant difference (*p* = 0.06).

### Effect of Lipidic Parameters on Platelet Aggregation

As the age-matched controls had mostly normal serum lipid levels but their platelet response values were higher than FH patients (Supplementary information Figure S3), we suppose that serum lipids can be responsible for the differences in platelet aggregation. Hence, in the last step, we correlated biochemical parameters with platelet aggregation data in the whole group of FH patients. The Pearson correlation coefficients are shown in Table 1. Highest correlation coefficients were observed for ristocetin. In all cases, platelet aggregation triggered by ristocetin increased with increasing levels of TC, LDL-C, non HDL-C and TG (Supplementary information Figure S5). No relationships to platelet aggregation were found for HDL-C and glucose. Other relationships were quite unexpectedly found mainly between TG and aggregation responses to AA and collagen (Supplementary information Figure S6).

**Table 1:** Relationships between serum lipids and different aggregation inducers with or without inhibitor

	AA 200 µM	AA 200 µM vs. ASA 30 µM	AA 200 vs. ASA 70 µM	collagen 1 µg/ml	collagen 1 µg/ml vs. ASA 70 µM	ADP 5 µM	ADP 5 µM vs. ticagrelor 0.5 µM	TRAP 10 µM	TRAP 10 µM vs. Vorapaxar 1 µM	ristocetin 400 µM	PAF 20 nM	U-46619 80 nM
LDL-C	0.05	0.07	− 0.14	0.11	0.13	0.12	− 0.26	− 0.01	0.22	0.48^*^	0.30	0.11
HDL-C	0.09	0.13	0.23	0.18	0.15	0.03	− 0.23	0.21	0.51^*^	0.19	0.22	− 0.02
TG	0.46^*^	0.46^*^	0.10	0.45^*^	0.59^**^	0.31	0.10	0.23	0.13	0.51^*^	− 0.03	− 0.08
TC	0.12	0.14	− 0.06	0.19	0.24	0.15	− 0.25	0.06	0.31	0.55^**^	0.29	0.09
Non HDL-C	0.11	0.12	− 0.11	0.16	0.21	0.15	− 0.22	0.02	0.22	0.53^**^	0.26	0.10
Glucose	− 0.13	− 0.07	0.06	− 0.24	0.04	0.02	0.30	− 0.20	− 0.14	− 0.12	− 0.19	0.03

Data are shown as the Pearson correlation coefficients. Bold values represent the highest and significant correlations. *p < 0.05, **p < 0.01. Inductors and inhibitors are shown at their final concentrations

## Discussion

In this study, we focused on the analysis of platelet function in patients suffering from familial hypercholesterolemia treated with lipid apheresis, but also with a relatively novel therapeutic option based on PCSK9ab. In addition, the impact of these treatments on platelet aggregation was compared with healthy volunteers. The main findings identified in this study were (i) PCSK9ab significantly decreased platelet aggregation and the effect was not inferior to apheresis, (ii) apheresis significantly decreased platelet response to collagen and ristocetin triggered aggregation, whereas PCSK9ab without apheresis achieved the same effect as apheresis with the exception of ristocetin, (iii) our FH treated patients had lower platelet reactivity to most inducers compared to age-matched healthy controls and (iv) low blood lipid level reduces the sensitivity to platelet inducers and thus increases the effectiveness of antiplatelet drugs, which was observed for ASA and vorapaxar.

Atherosclerosis and cardiovascular disease are very tightly related as long-term exposure to high lipid levels causes platelet hyperaggregability. LDL-C and platelet hyperfunction are interrelated because LDL-C is involved in activation and signal transduction in platelets, and therefore lowering blood lipids should reduce platelet activity. The treatment of FH is not simple in particular in relation to patients. There are several options often requiring pharmacological combinations to achieve the goal set by the European Society of Cardiology (ESC) [6, 26]. Statins are the most commonly prescribed drugs in lipid management, but only a small proportion of patients reach the target LDL-C level with maximum tolerated doses [6, 27]. Their effect in FH is rather based on protection against cardiovascular atherosclerotic events [28]. The combination of statins and ezetimibe is more advantageous and results in an about 15% reduction of LDL-C in FH [26, 29]. Moreover, lowering of LDL-C is more challenging in homozygous (HoFH) or severe heterozygous (HeFH) patients, and even the mentioned combination of statins with ezetimibe has often insufficient effect, making the use of a tri-combination necessary [27]. In HeFH patients, the ESC recommended the combination of statin at maximum tolerated doses and the addition of ezetimibe and a PCSK9ab. In HoFH patients, the treatment is enriched with LDL-apheresis [26]. In the present study, all HoFH and most HeFH patients received a tri-combination (statin + ezetimibe + PCSK9ab) and all HoFH patients also received LDL-apheresis (Supplementary information Table S5). In practice, LDL-apheresis is used in patients with HoFH and severe HeFH who respond poorly to pharmacological intervention [6]. Changes in blood LDL-C after lipid apheresis are around 70% [6–9] which is consistent with our data (Supplementary information Table S1). Our group has recently reported that lipid apheresis was found to reduce not only serum lipids, but biomarkers of inflammation as well [30]. The effects of LDL-apheresis on platelet function vary from study to study. Pares et al. demonstrated that the lowering of LDL-C influenced platelet aggregability triggered by ADP in a short term but the study was performed with just one patient [9]. In contrast to that finding, some other studies did not show the decreased aggregability immediately after apheresis [8, 31]. It is suggested that long-term LDL-apheresis has a more pronounced effect on platelet activity [7], because aggregability is influenced by LDL-C exposure over time. Lowering of LDL-C leads to normalization of platelet function [9]. These findings are consistent with our data. In this study, our patients have been treated for 16.5 years on average. Unfortunately, LDL-apheresis has some limitations such as re-elevation of LDL-C and certain discomfort because it is an invasive method [6]. Furthermore, the availability of this therapy is low in the Czech Republic, because lipid apheresis is carried out in just a few centres [32].

LDL-R is regulated by the serine protease PCSK9 and this is essential for the regulation of blood LDL-C. Excess of PCSK9 in blood leads to LDL-R degradation and elevates blood LDL-C. Moreover, PCSK9 binds to CD36 receptor on platelet surface and activates downstream signal transduction leading to enhanced aggregability [16]. Considering all these aspects, a positive impact of PCSK9ab was expected. Patients included in this study were treated with either alirocumab or evolocumab, both FDA/EMA approved PCSK9ab. These molecules bind specifically to PCSK9 and abolish its interaction with LDL-R [6]. PCSK9 inhibitors reduce LDL-C by 55–60% [33, 34] and, in combination with other hypolipidemics, this could reach a reduction of up to 85% [26]. Torres et al. compared the effect of LDL-apheresis and PCSK9ab, and their combination on LDL-C. The combination of these therapies decreased LDL-C numerically but did not bring a statistically significant improvement [35]. Regardless, PCSK9ab therapy is relatively new, brings a clear profit in terms of cholesterol reduction, but there are almost no data on its effect on platelets in metabolic diseases. In a study by Qi et al. [16], the benefit of evolucumab on aggregation was demonstrated. It was shown that aggregation is correlated with blood PSCK9 and high levels of PCSK9 increase hyperaggregability and evolucumab conversely reduced the potentiating effects of PCSK9 on platelets. However, enhanced expression of the gene for PCSK9 is not ordinary in FH [2]. Importantly, our results showed for the first time that therapy with antibodies against to PCSK9 decreased LDL-C as well as platelet aggregability. In addition, it should be mentioned that the heterozygous form of FH is not rare, its prevalence is estimated to be 1:200, so 40 million people worldwide are potentially at the risk of this disease and consequences. More patients could profit from this therapy because screening, diagnosis and awareness of FH in real population are insufficient [3].

In our study, pharmacotherapy with PCSK9 was not inferior in comparison to treatment with lipid apheresis in relation to platelet reactivity. In the case of TRAP, the pharmacotherapy group had even significantly lower platelet aggregability. This result should be however interpreted with care, as this was likely driven by the fact, that none of the FH patients in the pharmacotherapy group was of HoFH type. On the other hand, it seems that for most HeFH, which is a much more frequent form of FH, pharmacotherapy sufficiently decreases both LDL-levels and platelet hyperactivity. There was only one exception as patients in pharmacotherapy group reacted slightly more to ristocetin-induced aggregation in contrast to the post-apheresis group. The possible explanation can be that apheresis can decrease levels of von Willebrand factor, a pathway stimulated by ristocetin [36].

Current guidelines recommend lowering LDL-C below 2.6 mmol/L for moderate risk or below 1.8 mmol/L for high cardiovascular risk [6, 37]. Three patients out of a total six achieved LDL-C below 1.8 mmol/L in group before apheresis and in the pharmacotherapy group. However, immediately after apheresis, all six patients achieved LDL-C below 1.8 mmol/L. Quite interestingly, the closest relationship between aggregation induced by AA, collagen and ristocetin and metabolic parameters was observed for TG (Supplementary information Figures S5 and S6). Only three patients reached the recommend level of TG (< 1.7 mmol/L [37]) before apheresis with mean 1.4 ± 0.8 mmol/L. The pharmacotherapy group had similar TG with mean 1.5 ± 0.5 mmol/L and likewise three patients had TG below 1.7 mmol/L. TG-rich lipoproteins are also associated with hyperaggregability, although this is a rather less well-known fact compared to LDL-C [38–40]. Regardless, lowering both LDL-cholesterol and TG can linearly decrease platelet reactivity as we observed in our study.

### Limitation of Study

Inherently due to a low number of patients suffering with severe forms of FH, our study has some limitations. In particular, (1) the number of HoFH was only 4 due to very low prevalence of this human disease, (2) the treatment of FH is highly individual, and (3) some patients were administered regularly with antiplatelet drugs, and this complicated the interpretation of our results. Recruiting patients who undergo lipid apheresis and who do not take inhibitors of PCSK9 is a difficult task. Moreover, guidelines for FH patients recommend statins, ezetimibe, PCSK9 inhibitors [6, 27] and LDL-apheresis as an adjunctive tool [41] while other guidelines recommend statins, ezetimibe, bile acid-binding resins and LDL-apheresis [42]. Additionally, lipoprotein(a), which is also associated with FH [5], was not taken into account in this study.

## Conclusion

To our best knowledge, this is the first paper comparing the impact of different therapies in FH patients to their impact on platelet aggregation with many different platelet inducers. Additionally, the effect of these therapies was compared to age-matched generally healthy donors. This study demonstrated the benefit of PCSK9ab treatment in normalizing platelet function.

## Supplementary Information

Below is the link to the electronic supplementary material.

Supplementary file1 (DOCX 699 KB)

## Data Availability

We cannot share publicly the raw patient data, but they are available upon reasonable request to the corresponding author.

## Abbreviations

AA: Arachidonic acid
ADP: Adenosine-5-diphosphate
ASA: Acetylsalicylic acid
FH: Familial hypercholesterolemia
HDL-C: High-density lipoprotein cholesterol
HeFH: Heterozygous familial hypercholesterolemia
HoFH: Homozygous familial hypercholesterolemia
LDL-C: Low-density lipoprotein cholesterol
LDL-R: Low-density lipoprotein cholesterol receptor
non HDL-C: Non high-density lipoprotein cholesterol
PCSK9ab: Proprotein convertase subtilisin/kexin type 9 monoclonal antibodies
TC: Total cholesterol
TG: Triglycerides
TRAP: Thrombin receptor agonist peptide
