# Supplementary material for: The Impact of Convertase Subtilisin/Kexin Type 9 Monoclonal Antibodies with and without Apheresis on Platelet Aggregation in Familial Hypercholesterolemia
Source: Cardiovasc Drugs Ther. 2023 May 2;38(5):959–70. doi: 10.1007/s10557-023-07455-y (PMC11438737; doi:10.1007/s10557-023-07455-y)
Supplement: Supplementary file 1 — Supplementary file1 (DOCX 699 KB) [file 10557_2023_7455_MOESM1_ESM.docx]

**The impact of convertase subtilisin/kexin type 9 monoclonal antibodies with and without apheresis on platelet aggregation in familial hypercholesterolemia**

**Lukáš Konečný^1^, Marcel Hrubša^1^, Jana Karlíčková^2^, Alejandro Carazo^1^, Lenka Javorská^3^, Kateřina Matoušová^3^, Lenka Kujovská Krčmová^3, 4^, Vladimír Blaha^5^, Milan Bláha^5^, Přemysl Mladěnka^1*^**

^1^The Department of Pharmacology and Toxicology, Faculty of Pharmacy in Hradec Králové, Charles University, 50005 Hradec Králové, Czechia: [konecnylu@faf.cuni.cz](mailto:konecnylu@faf.cuni.cz); [hrubsam@faf.cuni.cz](mailto:hrubsam@faf.cuni.cz); [carazofa@faf.cuni.cz](mailto:carazofa@faf.cuni.cz); [mladenkap@faf.cuni.cz](mailto:mladenkap@faf.cuni.cz)

^2^The Department of Pharmacognosy and Pharmaceutical Botany, Faculty of Pharmacy in Hradec Králové, Charles University, 50005 Hradec Králové, Czechia: [karlickova@faf.cuni.cz](mailto:karlickova@faf.cuni.cz)

^3^The Department of Clinical Biochemistry and Diagnostics, University Hospital Hradec Králové, 50005 Hradec Králové, Czechia: [lenka.javorska@fnhk.cz](mailto:lenka.javorska@fnhk.cz); [katerina.matousova@fnhk.cz](mailto:katerina.matousova@fnhk.cz); [krcml1aa@faf.cuni.cz](mailto:krcml1aa@faf.cuni.cz)

^4^The Department of Analytical Chemistry, Faculty of Pharmacy in Hradec Králové, Charles University, 50005 Hradec Králové, Czechia

^5^The 3rd Department of Internal Medicine-Metabolic Care and Gerontology, University Hospital and Faculty of Medicine in Hradec Králové, Charles University, 50005 Hradec Králové, Czechia; [blaha@lfhk.cuni.cz](mailto:blaha@lfhk.cuni.cz); [blaham@email.cz](mailto:blaham@email.cz)

*Correspondence: [mladenkap@faf.cuni.cz](mailto:mladenkap@faf.cuni.cz)

SUPPLEMENTARY DATA

**15 pages**

**Table S1** Table of volunteers divided into groups according to treatment

|  | patients  (N) | median age  (range) | TC  (mmol/L) | LDL-C  (mmol/L) | BMI |
| --- | --- | --- | --- | --- | --- |
| all FH patients | 15 | 52 (40 – 77) | 4.30 ± 1.48 | 2.52 ± 1.38 | 29.15 ± 4.49 |
| undergoing apheresis | 8 | 56 (40 – 77) | 4.66 ± 1.70 | 2.72 ± 1.68 | 27.65 ± 4.21 |
| without apheresis | 7 | 50 (41 – 56) | 3.89 ± 1.06 | 2.29 ± 0.88 | 30.87 ± 4.17 |
| ^A^ PCSK9ab + before apheresis | 6 | 49 (40 – 77) | 4.73 ± 1.93** *^vs.^* ^C^ | 2.92 ± 1.89 * *^vs.^* ^C^ | 27.11 ± 4.72 |
| ^B^ PCSK9ab + after apheresis | - | - | 1.82 ± 0.55** *^vs.^* ^A,^ ** *^vs.^* ^C^ | 0.67 ± 0.49* *^vs.^* ^A,^ ** *^vs.^* ^C^ | - |
| ^C^ PCSK9ab without apheresis (pharmacotherapy group) | 6 | 51 (41 – 56) | 3.81 ± 1.12** *^vs.^* ^B^ | 2.22 ± 0.93** *^vs.^* ^B^ | 31.60 ± 4.08 |
| age-matched healthy donors | 15 | 59 (46 – 69) | 5.53 ± 0.78 | 3.46 ± 0.73 | 26.82 ± 3.29 |

Results are shown as average ± SD. **p*<0.05, ***p*<0.01, ****p*<0.001. P-values were calculated by a paired t-test /^A^before and ^B^after apheresis/ or an unpaired t-test /^A^ or ^B^ apheresis samples *vs.* ^C^pharmacotherapy group/. N: number of patients; TC: total cholesterol; LDL-C: low-density lipoprotein cholesterol; BMI: body-mass index, calculated with the known formula - weight [kg]/ (height [m])^2^.

**Table S2** Characterization of generally healthy control group and familial hypercholesterolemic patients.

|  |  | **Healthy donors** | **FH patients** | ***p*-value** |
| --- | --- | --- | --- | --- |
| patients, N (%) |  | 15 (100 %) | 15 (100 %) | - |
| age | 40-77 | 59 ± 6 | 52 ± 11 | 0.127 |
| BMI | 18.5-30+ | 29.27 ± 4.49 | 26.42 ± 3.29 | 0.128 |
| smokers | Yes | 6 (40 %) | 1 (7 %) | **0.031** |
| COVID-19^a^ | Yes | 7 (47 %) | 5 (33 %) | 0.456 |
| enteric-coated ASA | Yes | 0 (0 %) | 2 (13 %) | 0.143 |
| conventional ASA | Yes | 0 (0 %) | 3 (20 %) | 0.068 |
| clopidogrel + ASA combination | Yes | 0 (0 %) | 2 (13 %) | 0.143 |
| biochemical parameters | TC (mmol/L) | 5.53 ± 0.78 | 4.30 ± 1.48 | **0.010** |
|  | LDL-C (mmol/L) | 3.46 ± 0.73 | 2.52 ± 1.38 | **0.006** |
|  | glucose (mmol/L) | 5.45 ± 0.58 | 6.10 ± 2.05 | 0.336 |
|  | HDL-C (mmol/L) | 1.52 ± 0.43 | 1.18 ± 0.33 | **0.027** |
|  | TG (mmol/L) | 1.37 ± 0.45 | 1.81 ± 1.12 | 0.250 |
|  | creatinine in serum (µmol/L) | 80.54 ± 12.77 | 76.37 ± 17.22 | 0.314 |
|  | creatinine in urine (mmol/L) | 9.99 ± 4.17 | 10.86 ± 6.93 | 0.921 |

ASA: acetylsalicylic acid; BMI: body mass index; FH: familial hypercholesterolemia; HDL-C: HDL cholesterol; LDL-C: LDL cholesterol; N: number of patients; TC: total cholesterol; TGs: triglycerides. Data are shown as mean ± SD. Per cent values are related to the total number of subjects in the generally healthy donor group (n = 15) or FH patient group (n = 15). *p*-values were calculated using an unpaired *t*-test or chi-square test. Body mass index was calculated with the known formula: weight/ (height [m])^2^.

^a^ diagnosed with COVID-19 at different time points between 3 and 7 months prior to blood withdrawal.

**Table S3** Detailed characteristics of FH patients in subgroups

|  | |  | **undergoing apheresis**  **(apheresis group)** | **without apheresis**  **(pharmacotherapy group)** | ***p*-value** |
| --- | --- | --- | --- | --- | --- |
| patients, N (%) | |  | **6** | 6 | - |
| age | | 40-77 | 49 ± 13 | 51 ± 6 | 0.585 |
| BMI | | 18.5-30+ | 28.00 ± 4.72 | 32.32 ± 4.08 | 0.138 |
| smokers | | Yes | 0 (0 %) | 0 (0 %) | - |
| COVID-19^a^ | | Yes | 2 (33 %) | 3 (50 %) | 0.558 |
| Antithrombotic agents | enteric-coated ASA | Yes | 1 (16 %) | 1 (16 %) | 0.999 |
|  | conventional ASA | Yes | 2 (33 %) | 1 (16 %) | 0.505 |
|  | clopidogrel + ASA  combination | Yes | 1 (16 %) | 0 (0 %) | 0.296 |
| Lipid modifying agents | ezetimibe | Yes | 6 (100 %) | 6 (100 %) | - |
|  | lomitapide | Yes | 2 (33 %) | 0 (0 %) | 0.121 |
|  | a statin | Yes | 5 (83 %) | 6 (100 %) | **0.003** |
| CVD in detail | | arterial hypertension | 2 (33 %) | 0 (0 %) | 0.121 |
|  | | ACB | 1 (16 %) | 2 (33 %) | 0.505 |
|  | | AS of carotid arteries | 2 (33 %) | 2 (33 %) | 0.999 |
|  | | CAD | 3 (50 %) | 3 (50 %) | 0.999 |
|  | | PAD | 1 (16 %) | 0 (0 %) | 0.296 |
|  | | AS and calcifications / AS and defect / just AS of aortic valve | 3 (50 %) | 1 (16 %) | 0.221 |
|  | | stroke | 1 (16 %) | 0 (0 %) | 0.296 |
|  | | moderate stenosis of left ACC | 1 (16 %) | 0 (0 %) | 0.296 |
|  | | bilateral AS and calcifications of ACC | 1 (16 %) | 0 (0 %) | 0.296 |
|  | | haemodynamically not-significant Ao stenosis | 1 (16 %) | 0 (0 %) | 0.296 |
| diseases | | familial hypercholesterolemia | 6 (100 %) | 6 (100 %) |  |
|  | | diabetes mellitus type 1 | 1 (16 %) | 1 (16 %) | 0.999 |
|  | | hypothyreosis | 3 (50 %) | 0 (0 %) | **0.045** |
|  | | anaemia | 1 (16 %) | 0 (0 %) | 0.296 |
|  | | allergy | 1 (16 %) | 0 (0 %) | 0.296 |
|  | | glaucoma | 1 (16 %) | 0 (0 %) | 0.296 |
|  | | asthma | 1 (16 %) | 0 (0 %) | 0.296 |

ACB: aortocoronary bypass; ACC: arteria carotis communis; Ao: aortic; AS: atherosclerosis; BMI: body-mass index; CAD: coronary artery disease; CVD: cardiovascular disease; N: number of patients; PAD peripheral artery disease.

Data are shown as mean ± SD. Data are presented as numbers of both group of patients with the dominator of the percentage which is the total number of subjects in the group undergoing apheresis and in the pharmacotherapy group. *P*-values were calculated by an unpaired t-test or Chi-square test by GraphPad 9.3.1. BMI: body-mass index, calculated with the known formula - weight [kg]/ (height [m])^2^. Following drugs according to ATC code.

^b^diagnosed with COVID-19 at different time points between 3 to 7 months prior to blood withdrawal.

**Table S4** Summary of medications taken by the patients by ATC code

| **ANATOMICAL MAIN GROUP** | **THERAPEUTIC SUBGROUP** | **CHEMICAL SUBGROUP** | **Undergoing apheresis** | **Without apheresis** | ***p*-values** |
| --- | --- | --- | --- | --- | --- |
| A [**ALIMENTARY TRACT AND METABOLISM**](https://www.whocc.no/atc_ddd_index/?code=A&showdescription=no) | A02 [**DRUGS FOR ACID RELATED DISORDERS**](https://www.whocc.no/atc_ddd_index/?code=A02&showdescription=no) | A02BC [**Proton pump inhibitors**](https://www.whocc.no/atc_ddd_index/?code=A02BC&showdescription=no) | - | 1 | 0.269 |
|  | A07 [**ANTIDIARRHEALS, INTESTINAL ANTIINFLAMMATORY/ANTIINFECTIVE AGENTS**](https://www.whocc.no/atc_ddd_index/?code=A07&showdescription=no) | A07EC **[Aminosalicylic acid and similar agents](https://www.whocc.no/atc_ddd_index/?code=A07EC&showdescription=no)** | 1 | - | 0.333 |
|  | A10 [**DRUGS USED IN DIABETES**](https://www.whocc.no/atc_ddd_index/?code=A10&showdescription=no) | A10AE **[Insulins and analogues for injection, long-acting](https://www.whocc.no/atc_ddd_index/?code=A10AE&showdescription=no)** | - | 1 | 0.269 |
|  |  | A10BA **Biguanides** | 2 | 3 | 0.464 |
|  |  | A10BB **[Sulfonylureas](https://www.whocc.no/atc_ddd_index/?code=A10BB&showdescription=no)** | 1 | - | 0.333 |
|  |  | A10BD **[Combinations of oral blood glucose lowering drugs](https://www.whocc.no/atc_ddd_index/?code=A10BD&showdescription=no)** | - | - | 0.333 |
|  |  | A10BJ **[Glucagon-like peptide-1 (GLP-1) analogues](https://www.whocc.no/atc_ddd_index/?code=A10BJ&showdescription=no)** | 1 | - | 0.333 |
|  |  | A10BK [**Sodium-glucose co-transporter 2 (SGLT2) inhibitors**](https://www.whocc.no/atc_ddd_index/?code=A10BK&showdescription=no) | - | 1 | 0.269 |
|  | A12 [**MINERAL SUPPLEMENTS**](https://www.whocc.no/atc_ddd_index/?code=A12&showdescription=no) | A12AX **[Calcium, combinations with vitamin D and/or other drugs](https://www.whocc.no/atc_ddd_index/?code=A12AX&showdescription=no)** | 1 | - | 0.333 |
| B [**BLOOD AND BLOOD FORMING ORGANS**](https://www.whocc.no/atc_ddd_index/?code=B&showdescription=no) | B01 [**ANTITHROMBOTIC AGENTS**](https://www.whocc.no/atc_ddd_index/?code=B01&showdescription=no) | B01AA [**Vitamin K antagonists**](https://www.whocc.no/atc_ddd_index/?code=B01AA&showdescription=no) | 2 | - | 0.155 |
|  |  | B01AC **[Platelet aggregation inhibitors excl. heparin](https://www.whocc.no/atc_ddd_index/?code=B01AC&showdescription=no)** | 6 | 2 | 0.072 |
|  |  | B01AF [**Direct factor Xa inhibitors**](https://www.whocc.no/atc_ddd_index/?code=B01AF&showdescription=no) | 1 | - | 0.333 |
|  | B03 [**ANTIANEMIC PREPARATIONS**](https://www.whocc.no/atc_ddd_index/?code=B03&showdescription=no) | B03AE [**Iron in other combinations**](https://www.whocc.no/atc_ddd_index/?code=B03AE&showdescription=no) | 1 | - | 0.333 |
| C [**CARDIOVASCULAR SYSTEM**](https://www.whocc.no/atc_ddd_index/?code=C&showdescription=no) | C01 [**CARDIAC THERAPY**](https://www.whocc.no/atc_ddd_index/?code=C01&showdescription=no) | C01AA **[Digitalis glycosides](https://www.whocc.no/atc_ddd_index/?code=C01AA&showdescription=no)** | 1 | - | 0.333 |
|  |  | C01DA **[Organic nitrates](https://www.whocc.no/atc_ddd_index/?code=C01DA&showdescription=no)** | 1 | - | 0.333 |
|  | C03 [**DIURETICS**](https://www.whocc.no/atc_ddd_index/?code=C03&showdescription=no) | C03CA **[Sulfonamides, plain](https://www.whocc.no/atc_ddd_index/?code=C03CA&showdescription=no)** | 1 | - | 0.333 |
|  | C05 [**VASOPROTECTIVES**](https://www.whocc.no/atc_ddd_index/?code=C05&showdescription=no) | C05CA **[Bioflavonoids](https://www.whocc.no/atc_ddd_index/?code=C05CA&showdescription=no)** | 1 | - | 0.333 |
|  | C07 [**BETA BLOCKING AGENTS**](https://www.whocc.no/atc_ddd_index/?code=C07&showdescription=no) | C07AB [**Beta blocking agents, selective**](https://www.whocc.no/atc_ddd_index/?code=C07AB&showdescription=no) | 3 | 1 | 0.310 |
|  |  | C07AG **[Alpha and beta blocking agents](https://www.whocc.no/atc_ddd_index/?code=C07AG&showdescription=no)** | 1 | - | 0.333 |
|  | C08 [**CALCIUM CHANNEL BLOCKERS**](https://www.whocc.no/atc_ddd_index/?code=C08&showdescription=no) | C08CA **[Dihydropyridine derivatives](https://www.whocc.no/atc_ddd_index/?code=C08CA&showdescription=no)** | 2 | - | 0.155 |
|  | C09 [**AGENTS ACTING ON THE RENIN-ANGIOTENSIN SYSTEM**](https://www.whocc.no/atc_ddd_index/?code=C09&showdescription=no) | C09AA [**ACE inhibitors, plain**](https://www.whocc.no/atc_ddd_index/?code=C09AA&showdescription=no) | 3 | 2 | 0.714 |
|  |  | C09BB [**ACE inhibitors and calcium channel blockers**](https://www.whocc.no/atc_ddd_index/?code=C09BB&showdescription=yes) | 1 | - | 0.333 |
|  |  | C09BX [**ACE inhibitors, other combinations**](https://www.whocc.no/atc_ddd_index/?code=C09BX&showdescription=yes) | - | 1 | 0.269 |
|  | C10 [**LIPID MODIFYING AGENTS**](https://www.whocc.no/atc_ddd_index/?code=C10&showdescription=no) | C10AA [**HMG CoA reductase inhibitors**](https://www.whocc.no/atc_ddd_index/?code=C10AA&showdescription=no) | 7 | 7 | 0.333 |
|  |  | C10AB **[Fibrates](https://www.whocc.no/atc_ddd_index/?code=C10AB&showdescription=no)** | 1 | - | 0.333 |
|  |  | C10AX**[Other lipid modifying agents](https://www.whocc.no/atc_ddd_index/?code=C10AX&showdescription=no)** (C10AX09) | 7 | 7 | 0.333 |
|  |  | C10AX**[Other lipid modifying agents](https://www.whocc.no/atc_ddd_index/?code=C10AX&showdescription=no)** (C10AX12) | 2 | - | 0.155 |
|  |  | C10AX**[Other lipid modifying agents](https://www.whocc.no/atc_ddd_index/?code=C10AX&showdescription=no)** (C10AX13-14) | 6 | 6 | 0.605 |
|  |  | C10BA [**Combinations of various lipid modifying agents**](https://www.whocc.no/atc_ddd_index/?code=C10BA&showdescription=no) | 1 | - | 0.333 |
| G [**GENITO URINARY SYSTEM AND SEX HORMONES**](https://www.whocc.no/atc_ddd_index/?code=G&showdescription=no) | G04 [**UROLOGICALS**](https://www.whocc.no/atc_ddd_index/?code=G04&showdescription=no) | G04CA **[Alpha-adrenoreceptor antagonists](https://www.whocc.no/atc_ddd_index/?code=G04CA&showdescription=no)** | 1 | - | 0.333 |
| H [**SYSTEMIC HORMONAL PREPARATIONS, EXCL. SEX HORMONES AND INSULINS**](https://www.whocc.no/atc_ddd_index/?code=H&showdescription=no) | H03 [**THYROID THERAPY**](https://www.whocc.no/atc_ddd_index/?code=H03&showdescription=no) | H03AA **[Thyroid hormones](https://www.whocc.no/atc_ddd_index/?code=H03AA&showdescription=no)** | 3 | - | 0.070 |
| M [**MUSCULO-SKELETAL SYSTEM**](https://www.whocc.no/atc_ddd_index/?code=M&showdescription=no) | M04 [**ANTIGOUT PREPARATIONS**](https://www.whocc.no/atc_ddd_index/?code=M04&showdescription=no) | M04AA **[Preparations inhibiting uric acid production](https://www.whocc.no/atc_ddd_index/?code=M04AA&showdescription=no)** | 1 | - | 0.333 |
| N [**NERVOUS SYSTEM**](https://www.whocc.no/atc_ddd_index/?code=N&showdescription=no) | N05 [**PSYCHOLEPTICS**](https://www.whocc.no/atc_ddd_index/?code=N05&showdescription=no) | N05BA [**Benzodiazepine derivatives**](https://www.whocc.no/atc_ddd_index/?code=N05BA&showdescription=no) | 1 | - | 0.333 |
| R [**RESPIRATORY SYSTEM**](https://www.whocc.no/atc_ddd_index/?code=R&showdescription=no) | R03 [**DRUGS FOR OBSTRUCTIVE AIRWAY DISEASES**](https://www.whocc.no/atc_ddd_index/?code=R03&showdescription=no) | R03AK **[Adrenergics in combination with corticosteroids or other drugs, excl. anticholinergics](https://www.whocc.no/atc_ddd_index/?code=R03AK&showdescription=no)** | 1 | - | 0.333 |
|  |  | R03BA **[Glucocorticoids](https://www.whocc.no/atc_ddd_index/?code=R03BA&showdescription=no)** | 1 | - | 0.333 |

Drugs were divided according to appropriate ATC code to the groups by using ATC/DDD index 2022 by WHO. *P*-values were calculated by Chi-square test by GraphPad 9.3.1, all of them without statistical significance. ATC code of PCSK9ab is indicated in colour according to graphical scheme of the performed experiment.

**Table S5** Genetic and clinical data of recruited patients

|  |  |  | | GENETIC DATA |  |  |  |  | CLINICAL DATA |
| --- | --- | --- | --- | --- | --- | --- | --- | --- | --- |
| patient No.  (sex) | undergoing  apheresis (years) | | without apheresis | phenotype | gene | variant1  DNA changes | variant2  Effect of protein | exone location  No. | drugs |
| 1 (M) | - | | YES | HeFH | - | - | - | E4-8 | S+E+PCSK9ab |
| 2 (M) | - | | YES | HeFH | LDL-R | - | p.Asp345Ala | E9 | S+E+PCSK9ab |
| 3 (F) | - | | YES | HeFH | LDL-R | - | p.Gln378Pro | E8 | S+E+PCSK9ab |
| 4 (M) | - | | YES | HeFH | LDL-R | - | p.Gly324AlafsX46 | E7 | S+E+PCSK9ab |
| 5 (M) | - | | YES | HeFH | LDL-R | c.2072C>A | p.Ser691Ter | - | S+E+PCSK9ab |
| 6 (M) | - | | YES | HeFH | LDL-R | c.2072C>A | p.Ser691Ter | - | S+E+PCSK9ab |
| 7 (M) | - | | YES | HeFH | LDL-R | c.1775G>A | p.Gly592Glu | E12 | S+E |
| 8 (F) | YES (10) | | - | HoFH | LDL-R | - | p.Asp354Ala | E8 | S+E+PCSK9ab |
| 9 (F) | YES (22) | | - | HeFH | - | - | - | - | S+E+PCSK9Ab |
| 10 (M) | YES (5) | | - | HeFH | - | EX9_14del | - | E9-14 | E+PCSK9ab |
| 11 (F) | YES (19) | | - | HeFH | - | - | - | - | S+E |
| 12 (M) | YES (25) | | - | HeFH | - | - | - | - | S+Fi |
| 13 (M) | YES (20) | | - | HoFH | LDL-R | c.1775G>A; c.2390-1G>A | - | E12; E16 | S+E+L+PCSK9ab |
| 14 (F) | YES (5) | | - | HoFH | LDL-R | c.1729T>G; c.1414G>T | p.Trp577Gly p.Asp472Tyr | - | S+E+PCSK9ab |
| 15 (F) | YES (22) | | - | HoFH | LDL-R | c.1474G>A; c.1775G>A | - | E10; E12 | S+E+L+PCSK9ab |

F: female; HeFH: heterozygous familial hypercholesterolemia; HoFH: homozygous familial hypercholesterolemia; LDL-R: low density lipoprotein receptor; M: male; No.: number. Following drugs according to ATC code E: ezetimibe (C10AX09); Fi: fenofibrate (C10AB); L: lomitapide (C10AX12); PCSK9ab: convertase subtilisin/kexin type 9 monoclonal antibodies (C10AX13-14); S: statins (C10AA).

**Table S6** Biochemical parameters.

|  | | **p-value^A^**  **pre *vs.* post apheresis** | **p-value^B^**  **post apheresis *vs.* drug treated group** |
| --- | --- | --- | --- |
| biochemical parameters | LDL cholesterol [mM] | **0.019** | 0.479 |
|  | HDL cholesterol [mM] | **0.005** | 0.155 |
|  | triglycerides [mM] | **0.011** | 0.707 |
|  | total cholesterol [mM] | **0.010** | 0.378 |
|  | creatinine in serum [µM] | 0.434 | 0.880 |
|  | creatinine in urine [mM] | **-** | 0.085 |
|  | glucose [mM] | 0.215 | 0.394 |

Data are related to subjects in the groups undergoing apheresis (n = 6) and the without-apheresis group (n = 6). *p*-values were calculated using a paired *t*-test /^A^ before and after apheresis/or an unpaired *t*-test /^B^ apheresis sample *vs.* pharmacotherapy group/. Creatinine in urine was measured only before the apheresis procedure.

**Table S7** Summary of inducers and inhibitors.

|  | | **final concentration** | **units** |
| --- | --- | --- | --- |
| inducers | collagen | 1 | µg/mL |
|  | arachidonic acid (AA) | 200 | µM |
|  | ristocetin | 400 | µM |
|  | platelet activating factor-16 (PAF) | 20 | nM |
|  | U-46619 | 80 | nM |
|  | adenosine-5-diphosphate (ADP) | 5 | µM |
|  | thrombin receptor activating protein-6 (TRAP) | 10 | µM |
| inhibitors | acetylsalicylic acid (ASA) | 30; 70 | µM |
|  | ticagrelor | 0.5 | µM |
|  | vorapaxar | 1; 5 | µM |

**Figure S1** Biological parameters in all PSCK9ab-treated patients with or without apheresis **A:** LDL-cholesterol, **B:** HDL-cholesterol. **C:** triglycerides. **D:** total cholesterol**. E:** non-HDL-cholesterol. *P*-values were calculated by a paired test /pre- and post-apheresis/ or an unpaired test /apheresis samples *vs.* pharmacotherapy group/. Results are shown as median with 95% confidence interval.

**Figure S2** Comparison of the remaining inducers of platelet aggregation in all PCSK9ab-treated patients with or without apheresis **A:** AUC of aggregation induced by AA at a final concentration of 200 µM. **B:** AUC of aggregation induced by ADP at a final concentration of 5 µM. **C:** AUC of aggregation induced by PAF at a final concentration of 20 nM. **D:** AUC of aggregation induced by U-46619 at a final concentration of 80 nM. *P*-values were calculated by a paired test /pre- and post-apheresis/ or an unpaired test /apheresis samples *vs.* pharmacotherapy group/. Results are shown as median with 95% confidence interval.

**Figure S3** Biological parameters in group of healthy donors and familial hypercholesterolemia patients (FH, all 15 patients) **A:** LDL-cholesterol. **B:** HDL-cholesterol. **C:** triglycerides. **D:** total cholesterol. **E:** non-HDL-cholesterol. *P*-values were calculated by an unpaired test /samples of healthy donors *vs.* treated patients. Results are shown as median with 95% confidence interval.

**Figure S4** Comparison of the influence of inhibitors to platelet aggregation in all PCSK9ab-treated patients with or without apheresis **A:** AUC of aggregation induced by AA in blood pretreated with 30 µM ASA. **B:** AUC of aggregation induced by AA in blood pretreated with 70 µM ASA. **C:** AUC of aggregation induced by collagen in blood pretreated with 70 µM. **D:** AUC of aggregation induced by ADP in blood pretreated with 0.5 µM ticagrelor. *P*-values were calculated by a paired test /pre- and post-apheresis/ or an unpaired test /apheresis samples *vs.* pharmacotherapy group/. Results are shown as median with 95% confidence interval.

 **Figure S5** The effect of blood lipids on platelet aggregation induced by ristocetin **A:** ristocetin and LDL-C. **B:** ristocetin and TG. **C:** ristocetin and TC. **D:** ristocetin and non-HDL-C. *P*-values were calculated by the Pearson correlation analysis, graphs were constructed by a simple linear regression.

 **Figure S6** Other significant associations between biochemical parameters and platelet aggregation **A:** TG and AA. **B:** TG and the inhibitory response to ASA on AA triggered aggregation. **C:** TG and collagen. **D:** TG and the inhibitory response to ASA on collagen triggered aggregation. *P*-values were calculated by Pearson correlation analysis, graphs were constructed by a simple linear regression.

**Table S8** Summary of familial hypercholesterolemia patients after exclusion of patients treated with conventional antiplatelet therapy and all patients treated with any antiplatelet therapy.

| inductor | inhibitor | p-value^a^  healthy *vs.* FH not-treated by any conventional antiplatelet therapy | p-value^b^  healthy *vs.* FH administered with no antiplatelet therapy |
| --- | --- | --- | --- |
| AA 200 µM | - | **p = 0.002^c^** | **p = 0.009^c^** |
| AA 200 µM | ASA 30 µM | **p = 0.035^c^** | p = 0.102 |
| AA 200 µM | ASA 70 µM | **p = 0.049^c^** | p = 0.092 |
| collagen 1 µg/ml | - | **p = 0.005^c^** | **p = 0.023^c^** |
| ADP 5 µM | - | p = 0.062 | p = 0.124 |
| ADP 5 µM | ticagrelor 0.5 µM | p = 0.999 | p = 0.740 |
| ristocetin 400 µM | - | **p = 0.001^c^** | **p = 0.012^c^** |
| U-46619 80 nM | - | **p = 0.040^c^** | p = 0.053 |
| TRAP 10 µM | vorapaxar 1 µM | **p = 0.034^c^** | **p = 0.021^c^** |

AA: arachidonic acid; ADP: adenosine-5-diphosphate; ASA: acetylsalicylic acid: FH: familial hypercholesterolemia; TRAP: thrombin receptor activating protein-6.

^a^Data are presented as total number of subjects in the healthy group (n=15) and in familial hypercholesterolemia group with no conventional antiplatelet therapy (n=10).

^b^Data are presented as total number of subjects in the healthy group (n=15) and in familial hypercholesterolemia group with no antiplatelet therapy (n=8).

*P*-values were calculated by an unpaired t-test. Bold values represent the significant changes. Inductors and antagonists/inhibitors are reported in their final concentrations. ^c^The platelet aggregation response was lower in familial hypercholesterolemia group.

References:

WHOCC-ATC/DDD index 2023 [online]. [cit. 2023-01-10]. From <https://www.whocc.no/atc_ddd_index/>
